# Evaluating and comparing tolerance, nutritional quality and bio-functional activity of bovine-plasma, corn and whey proteins, outcomes of a randomized double blind controlled trial

**DOI:** 10.1016/j.crfs.2023.100588

**Published:** 2023-09-12

**Authors:** Diederik Esser, Ron Wehrens, Kaatje Lenaerts, Jasper Engel, Ronald T.M. van den Dool, Shanna Bastiaan-Net, Jurriaan J. Mes, Harry J. Wichers

**Affiliations:** a Wageningen Food & Biobased Research, Wageningen University & Research, Wageningen, the Netherlands; b Department of Surgery, NUTRIM School of Nutrition and Translational Research in Metabolism, Maastricht University, Maastricht, the Netherlands; c Wageningen Biometris, Wageningen University & Research, Wageningen, the Netherlands

**Keywords:** Dietary protein, Protein concentrates isolates, Tolerance, Health, Nutrient quality

## Abstract

Important considerations in the choice of future sustainable protein sources for human application are tolerance, nutritional quality, and potential health benefits. We evaluated, in a double-blind cross-over intervention trial, tolerance, nutritional quality, and potential health effects of two sustainable protein sources. Thirty-six apparently healthy older adults (age 62.3 ± 7.2yrs, BMI 25 ± 3 kg/m2) received 40 g/day bovine-plasma protein (BP), corn protein (CP) or, as a benchmark, whey protein (WP) for one week with a washout period of one week in-between. In 12 participants, we also determined postprandial amino acid (PAA) uptake kinetics upon consumption of 20 g BP, CP, or WP. Changes in self-reported gastrointestinal complaints and intestinal permeability assessed using a multi-sugar acetylsalicylic acid test did not differ between the interventions. Clear differences in PAA responses were observed after consumption of the different proteins, but clear essential amino acid responses were observed for all proteins. BP consumption resulted in a small but significant increase in blood pressure outcomes, and CP consumption resulted in a small but significant decrease in insulin levels when compared to the other interventions. In conclusion, alternative protein concentrates and isolates studied here can be consumed in relative high quantities without experiencing unwanted GI complaints or gut barrier dysfunction and they can be a good source of essential amino acids. The rise in blood pressure observed during the BP intervention, potentially linked to the elevated salt content of the BP, constitutes a potential health issue. Future studies with longer intervention periods might however be recommended.

## Highlights

• Proteins were consumed in relative high quantities without experiencing unwanted GI complaints or gut barrier dysfunction
• The studied proteins (corn and bovine-plasma) can be a good source of essential amino acids
• Future studies focusing on potential health effects are recommended

## Graphical abstract

**Figure:**
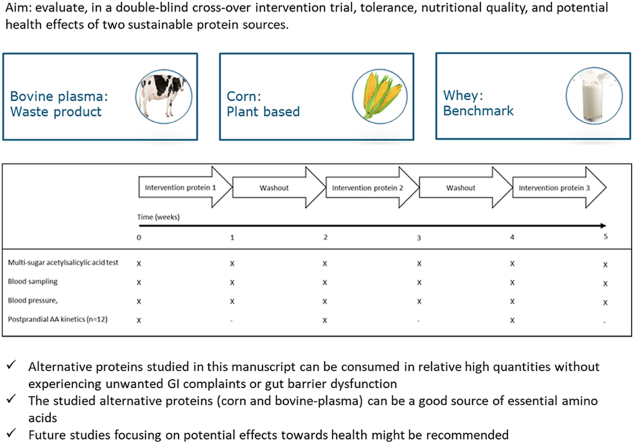

## 1 Introduction

In the forthcoming years, a dramatic transition in the supply of protein for human nutrition is anticipated. Due to global population growth, prosperity and demographic changes, a considerable increase in the demand for protein is expected (Gilland 2002). As sustainability considerations prohibit that this increasing protein demand can be met by conventional animal sources, we need to shift to a larger share of sustainably produced proteins for inclusion in the human diet. Thus, there is an urgent need for sustainable, safe, healthy and resource-efficiently produced proteins for inclusion in the human diet. Next to focusing on plants-derived proteins, we also need to deal efficiently with all unused by-products. By-products including those derived from animal sources, are often used as feed, while these products could perhaps be better disposed of in the food domain after minimal processing steps. Many of these new sustainable proteins are included in food products as protein isolates or concentrates, facilitating its use in a wide range of foods. Alternative isolated proteins are for instance used in meat analogues, a growing novel food category, of which the knowledge regarding their impact on human health is very limited. We need to know if we can consume relatively high amounts of these alternative protein concentrates and/or isolates on a regular basis, while safeguarding nutritional requirements in terms of amino acid and bioactive peptides supply or without negative health impacts. Although proteins share common features, no generic criteria are yet known for predicting their nutritional functionality. The most important nutritional role of dietary protein is providing human metabolism with amino acids for incorporation into endogenously produced proteins. Particularly their content in essential amino acids, as well as their digestibility and bioavailability are important quality parameters. A second nutritional/physiological role of dietary protein is that they are a source of, transiently present, peptides with biological functions such as impact on the epithelial barrier, regulation of appetite and blood pressure (Sharkey et al., 2020; Bao and Wu 2021, Chelliah et al., 2021). So taken together, when considering alternative protein isolates for human consumption, we need to evaluate their tolerance, nutritional quality, and potential health effects.

We selected two alternative protein sources for inclusion in this study. These were derived from the plant source corn and from the by-product bovine plasma. Corn has a high yield potential and is a major cereal crop in the world. Protein quality and quantity of corn has been improved over the last decades due to successful bio-fortification (Zhu et al., 2019). The use of animal blood plasma as food is safe and subjected to strict EC food regulations and with versatile interesting food applications (Lynch et al., 2017). Animal blood coming from slaughterhouses represents the most problematic by-product of the meat industry because of the high volumes routinely generated globally (Lynch et al., 2017). A previous study also indicated that bovine blood derived protein can contain bioactive peptides (Zhang et al., 2015). Whey protein was selected as reference due to its complete amino acid profile and rapid digestion and absorption. Its rich composition of essential amino acids and quick delivery to the body set a valuable standard for evaluating other protein sources. Moreover, the established use of whey protein as a reference in numerous studies enables the contextualization of results within existing scientific literature.

In the current study, we aimed to investigate tolerance, nutritional quality, and potential health effects of bovine-plasma protein (BP) and corn protein (CP) consumption in a healthy (older) adult population and to investigate how these aspects relate to the commonly consumed benchmark protein whey protein (WP). We did this by determining the impact on gut-barrier function, gastrointestinal complaints and cardiometabolic health after daily consumption of 40 g of protein for a week, as well as through measuring postprandial amino acid uptake kinetics after a bolus of 20 g of proteins.

## 2 Subjects and methods

### 2.1 Ethics statement

This study has been reviewed and approved by the Medical Ethics Committee of Wageningen, The Netherlands. Furthermore, the study was conducted according to the principles of the Declaration of Helsinki, in accordance with the Medical Research Involving Human Subjects Act (WMO) and registered at Clinical Trials.gov (identifier NCT03744221). All subjects gave their written informed consent before entering the study.

### 2.2 Subjects

Thirty-six apparently healthy adults (n = 17 men and 19 women) with an age range between 30 and 70 years (mean ± SD = 62.3 ± 7.2 years) and a BMI between 18.5 and 24.9 kg/m2 were recruited from the surroundings of Wageningen. All subjects were non-smoking, did not have any metabolic, gastrointestinal, inflammatory or chronic disease, or a history of gastro-intestinal surgery or (serious) gastro-intestinal complaints.

### 2.3 Study design

The study was a double blind, randomized, cross-over, controlled trial consisting of three different protein interventions. Study subjects received three different protein sources of 40 g/day for one week with a washout period of one week between interventions. They visited our research facility before and after each intervention period on two separate occasions to measure gut permeability via a multi-sugar test after an acetylsalicylic acid challenge (test day 1), to collect fasting blood samples and blood pressure outcomes, and to perform a pulse wave analysis (test day 2).

**Fig. 1:**
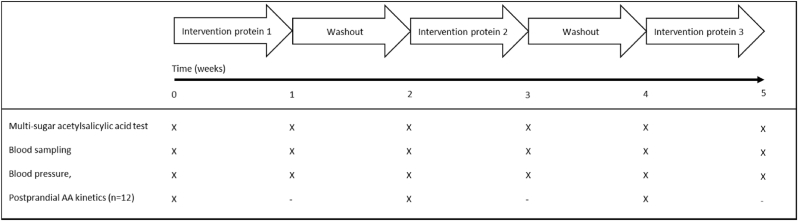
Schematic overview of short-term intervention plan and points and sort of measurements.

A subset of 12 volunteers, selected from the initial study population, was subjected to a protein PAA absorption kinetics protocol at the start of each intervention period. An overview of the measured outcomes in the study design is depicted in Fig. 1.

On the day prior to all study days, participants received a standardized meal and were instructed not to eat or drink anything except water after 08.00 p.m. The participants were also instructed not to drink alcohol or to perform heavy exercise the day prior to a study day. Study subjects were instructed by a dietician not to lose or gain weight during the entire intervention, which was checked weekly.

### 2.4 Intervention products

Study subjects consumed 40 g of the test proteins a day during each intervention, on top of their normal daily protein intake. Proteins were standardized consumed on two occasions, e.g. before lunch and dinner. Protein content of BP was 70% (Sonac Plasma Powder 70 B, Sonac Loenen BV, Loenen, The Netherlands) CP 85% (corn protein isolate, Cargill, Vilvoorde, Belgium) and WP 80% (whey protein concentrate 80 L^02^, Milei, Leutkirch, Germany). The amount of product the study subjects consumed was therefore slightly adapted to standardize the amount of consumed protein; 57 g, 47 g and 50 g for respectively BP, CP and WP (Supplemental Table 1). Proteins were dissolved in 100 ml commercially available vegetable juice and 200 ml of tap water, in order to improve blinding and improve dissolving, still protein drinks differed in taste. Proteins consumed for the measurement of PAA kinetics were dissolved in water.

### 2.5 Test day 1: multi-sugar test after acetylsalicylic acid challenge

The procedure was based on van Wijck et al. (van Wijck et al., 2013; Hoshiko et al., 2021). On the day prior to each gut permeability test, participants were instructed to avoid spicy food and products containing sucralose or erythritol. In the evening prior to the gut permeability test, study subjects took orally 1000 mg of acetylsalicylic acid (2 tablets of 500 mg aspirin, Bayer, Germany) with a full glass of water. The next morning, 1 h before the consumption of the multi-sugar (MS) mix, subjects again took 1000 mg of acetylsalicylic acid with a full glass of water.

After emptying their bladder, study subjects consumed the MS mix containing five sugars; 1 g sucrose (van Gilse, Dinteloord, the Netherlands), 1 g lactulose (Lactulose CF, Centrafarm, Etten-Leur, the Netherlands), 0.5 g L-rhamnose (L-rhamnose monohydrate crystalline, Danisco/DuPont, Dordrecht), 1 g sucralose (SPLENDA© Sucralose granular DFF-1, Brenntag, Dordrecht) and 1 g erythritol (Zerose^TM^Erythritol 16,952, Cargill, Amsterdam) dissolved in 200 ml tap water. From that point onwards, all urine was collected for 24 h (h). The first 0–5 h fraction, representing small intestinal permeability, was collected at the research facility under fasting conditions. The remaining period, urine was collected at home at room temperature. This 5–24 h urine collection, representing colon permeability, was handed over to the researchers the next day, when they arrived for their second test day.

### 2.6 Test day 2: blood collection and blood pressure measurement

At the start of test day 2, study subjects handed over their 5–24 h urine sample that was collected at home. Next, blood pressure measures were performed after 10 min of rest. Systolic blood pressure (sBp), diastolic blood pressure (dBp) and heart rate (HR) were assessed automatically (DINAMAP® PRO 100) for 10 min with a 3-min interval. A pressure sensor (applanation tonometer) was applied on the radial artery to record pulse pressure waveforms as previously described (Esser et al., 2013). The waveform was calibrated using systolic and diastolic pressure values from the conventional cuff measurement. From these peripheral waveforms, an aortic pressure waveform was derived and a pulse wave analysis (PWA) was conducted. PWA was subsequently used to identify aortic blood pressures and the Augmentation Index (Aix), a measure of arterial stiffness (Laurent et al., 2006). After the blood pressure measurements we collected the blood samples.

On the second test day and prior to the intervention period, twelve participants received a postprandial test with the protein source they had to consume during that intervention period. A cannula was inserted in the arm of these participants, and after a fasting blood collection, the participant consumed an equivalent of 20 g protein (23.5 g CP, 27.5 g BP and 25 g WP) within a time frame of 10 min. Postprandial blood samples were collected from the cannula 15, 30, 45, 60, 75, 90, 120, 150 and 180 min after intake of the protein source. These 12 subjects had to consume the other 20 g test protein for that day before dinner to match the daily 40 g of test protein intake.

### 2.7 Clinical chemistry

Plasma glucose and insulin were analysed by a hospital laboratory (ZGV, Ede, The Netherlands).

Blood derived free amino acid analysis.

Free amino acids in collected blood were analysed as were described previously (Mes et al., 2021) and based on the Waters AccQ Tag method for amino acid analysis.

### 2.8 Urinary multi-sugar analysis

Urine sugar concentrations were determined by isocratic ion exchange high-pressure liquid chromatography (Model PU-1980 pump; Jasco, Easton, MD, USA) with mass spectrometry (Model LTQ XL; Thermo Fisher Scientific, Waltham, MA, USA) as described by van Wijck et al. (van Wijck et al., 2011).

### 2.9 Statistical analysis

The time curves for PAA levels in blood of individual participants were fitted by using an R-package specifically designed for analyzing amino acid response curves in cross-over intervention studies as previously described (Mes et al., 2021). The area under the curve (AUC), peak height and time-to-peak (time2max) were compared in an ANOVA analysis to test the null hypothesis over overall equality, with a post-hoc test to identify where any differences are located. As a second type of analysis, a linear mixed model was used to specifically focus on the differences between WP as a reference, and the other two proteins. In all cases, a P value of 0.05 was used as the threshold of statistical significance.

Statistical analysis on the changes before and after the intervention was performed by linear mixed models for repeated measures (PASW Statistics 25; IBM SPSS, Chicago, IL, USA). Analysis was performed by using protein treatment (P), time in weeks (T), and the interaction treatment × time (I) as fixed effects. The time effect (T) indicates whether there is a change over time, regardless of the intervention given. A protein treatment (P) effect indicates a difference between the protein interventions, but does not account for the change over time (baseline values are included in this case). The interaction treatment × time (I) effect indicates if the change within the intervention period is different between the protein sources. In all cases a P value of 0.05 was used as the threshold of statistical significance.

## 3 Results

### 3.1 Study logistics

We recruited participants and assessed 61 individuals for eligibility. Twenty-five individuals were excluded because they did not meet our inclusion criteria or withdrew themselves. Thirty-six persons were included in the study and randomly allocated to a treatment order. All 36 subjects completed the intervention study. The baseline characteristics of these 36 participants and of the 12 participants subjected to the protein PAA uptake kinetic protocol are summarized in Table 1. Compliance with the intervention was based on percentages of product packages returned combined with self-reported intake via daily on-line questionnaires. Based on these outcomes, overall compliance was 99%. Study participants did not significantly loose or gain weight during the intervention period.

**Table 1:** Baseline characteristics of the 36 subjects that finalised the whole study protocol and of the 12 participants that received a PAA uptake test.

	Subjects whole study (n = 36)	Sub group postprandial test (n = 12)
Males/females	17/19	4/8
Age (y)	62 ± 8	62 ± 6
BMI (kg/m^2^)	25 ± 3	24 ± 3

Values are expressed as Mean ± SD.

### 3.2 GI complaints and stool patterns

Changes in reported GI complaints during the one-week protein intervention did not differ between BP, CP and WP (Table 2, P-value >0.05 for interaction effect protein × time). In general, participants scored significantly higher ratings for belching and nausea when consuming the BP, and these scores did not change over the 7-day intervention period (P-value = 0.03 and < 0.01 respectively for protein effect). Lowest appreciation scores (VAS ranging from 0 to 10) were reported for BP (3.5 ± 1.9), followed by CP (4.3 ± 1.6) and WP (5.7 ± 1.2). These scores differed significantly (P < 0.01) and did not change over the 7-days intervention period (P = 0.78 for interaction effect protein × time). The number of stools and stool consistency remained stable over all intervention periods (Supplemental Figs. 1 and 2). The average number of stools during the intervention was 1 defecation per day and the majority of stools were classified as type 3, 4 or 5 on the Bristol stool chart. All stool patterns were stable and fitted in a healthy stool pattern during the entire study.

**Table 2:** Daily reported GI complaints during a one-week BP, CP or WP protein intervention.

		Day							P-value*		
		**1**	**2**	**3**	**4**	**5**	**6**	**7**	T	P	I
Bloating	BP	11.7 ± 18.3	14.5 ± 20.3	13.3 ± 17.2	13.1 ± 19.8	11.9 ± 16.7	11.2 ± 17.0	13.3 ± 17.4			
	CP	14.0 ± 21.4	10.5 ± 1.1	11.9 ± 18.3	12.4 ± 19.1	14.3 ± 21.4	14.0 ± 22.7	13.1 ± 20.2	0.82	0.44	0.84
	WP	15.8 ± 21.4	13.9 ± 20.1	15.3 ± 20.2	12.9 ± 22.4	12.4 ± 19.0	11.1 ± 17.6	11.6 ± 17.6			
Belching	BP	22.0 ± 25.4	19.6 ± 24.5	19.5 ± 21.2	15.8 ± 18.9	18.6 ± 22.7	13.8 ± 18.2	17.4 ± 23.8			
	CP	11.9 ± 20.4	9.6 ± 15.6	13.1 ± 19.7	12.2 ± 21.3	12.6 ± 21.2	12.6 ± 21.6	12.4 ± 20.1	0.52	0.03	0.64
	WP	10.5 ± 16.8	13.6 ± 20.6	10.6 ± 15.8	10.1 ± 14.7	9.4 ± 15.1	8.4 ± 14.5	9.9 ± 15.0			
Flatulence	BP	10.7 ± 15.2	17.5 ± 21.4	20.7 ± 20.1	19.5 ± 20.5	19.1 ± 19.5	21.2 ± 22.1	18.1 ± 19.7			
	CP	13.1 ± 18.9	11.4 ± 17.5	18.4 ± 22.7	15.9 ± 22.3	18.3 ± 21.3	15.8 ± 2.3	14.1 ± 20.4	0.16	0.49	0.75
	WP	13.4 ± 14.9	17 ± 21	17.1 ± 21.5	18.4 ± 22.7	17.2 ± 19.7	19.6 ± 22.4	17.4 ± 18.8			
Nausea	BP	14.7 ± 25.5	12.9 ± 18.2	13.1 ± 18.6	14.2 ± 24.1	11.4 ± 21.3	6.8 ± 10.7	11.9 ± 22.1			
	CP	9.6 ± 20.2	9.2 ± 20.5	9.0 ± 15.7	11.9 ± 19.0	8.6 ± 15.1	7.1 ± 15.0	4.6 ± 7.3	0.40	<0.01	0.91
	WP	4.7 ± 6.2	4.6 ± 9.3	4.7 ± 6.6	4.8 ± 6.4	5.1 ± 10.8	3.7 ± 3.8	4.6 ± 8.5			
Stomach-ache	BP	6.1 ± 8.0	6.4 ± 8.8	6.3 ± 6.8	6.7 ± 12.4	11.8 ± 21.3	8.8 ± 18.6	8.5 ± 14.0			
	CP	10.9 ± 23.9	6.3 ± 12.3	9.7 ± 18.2	10.1 ± 15.4	11.8 ± 19.6	13.5 ± 26.2	7.9 ± 16.4	0.69	0.29	0.40
	WP	8.6 ± 13.9	7.6 ± 13	12 0 ± 27.1	7.4 ± 12.6	4.2 ± 5.4	4.2 ± 4.5	5.5 ± 9.9			
Diarrhea	BP	6.1 ± 13.9	9.2 ± 19.1	8.4 ± 18.6	6.2 ± 13.5	9.1 ± 18	4.4 ± 6.0	3.7 ± 4.1			
	CP	4.8 ± 12.1	9.3 ± 19.9	6.8 ± 13.2	3.8 ± 3.5	5.8 ± 9.2	6.5 ± 14.2	5.1 ± 11.6	0.13	0.67	0.85
	WP	6.2 ± 14.4	6.5 ± 16	9.0 ± 17.9	5.5 ± 10.2	3.7 ± 4.2	5.1 ± 10.4	3.1 ± 3.1			
Constipation	BP	6.6 ± 8.6	5.6 ± 7.3	7.3 ± 15.9	4.1 ± 4.7	5.0 ± 7.6	5.9 ± 11.0	6.7 ± 10.9			
	CP	3.4 ± 3.3	5.3 ± 15.9	7.0 ± 16.8	4.9 ± 6.9	7.5 ± 19.6	4.8 ± 8.1	7.4 ± 12.4	0.28	0.08	0.41
	WP	8.2 ± 14.0	10.4 ± 18.3	5.0 ± 6.2	7.1 ± 12.7	6.8 ± 11.0	6.9 ± 10.4	14.1 ± 20.6			

Mean ± SD (n = 36)., *calculated by using linear mixed models. Outcomes were self-assessed scores reported on a Visual Analogue Scale (VAS) ranging from ‘not at all’ (0) to ‘a lot’ (100) presence. BP: bovine plasma, CP: corn protein, WP: whey protein. T = effect of time, P = effect of protein treatment, I = interaction time x protein treatment.

### 3.3 Gut permeability

Table 3 lists the effects of one week daily consumption of either BP, CP or WP protein on intestinal permeability assessed using a multi-sugar acetylsalicylic acid test. Changes in urinary ratios before and after one week daily consumption did not differ between protein interventions (P-value >0.05 for interaction effects). Protein interventions did decrease urinary sucrose/rhamnose ratios from 0 to 5 h samples collection, but this decrease was mainly due to a relative high baseline values in the third intervention week, observed for all three interventions (Supplemental Table 2). Protein interventions increased urinary sugar ratios sucralose/erythritol, but these responses did not differ between the BP, CP and WP interventions.

**Table 3:** Urinary sugar ratios after an acetylsalicylic acid challenge, before and after a one-week CP, BP or WP intervention.

Location permeability	Sugar markers (x10^3^)	Protein intervention								
		BP		CP		WP		P-value*		
		before	after	before	after	before	after	T	P	I
Gastroduodenum	Sucrose/rhamnose (F1)^1^	21 ± 24	15 ± 17	21 ± 23	15 ± 17	15 ± 19	11 ± 9	0.01	0.36	0.88
Small intestine	Lactulose/rhamnose (F1)^2^	67 ± 50	63 ± 37	66 ± 34	72 ± 45	64 ± 35	62 ± 32	0.62	0.26	0.22
Colon	Sucralose/erythritol (F2)^3^	20 ± 7	26 ± 6	19 ± 8	24 ± 8	19 ± 6	25 ± 6	<0.01	0.43	0.69
Whole intestine	Sucralose/erythritol (F1-2)^3^	19 ± 7	25 ± 6	18 ± 7	24 ± 7	18 ± 6	25 ± 7	<0.01	0.61	0.96

Mean ± SD, *calculated by using linear mixed models. F1: urinary fraction 0–5 h, F2: urinary fraction 5–24 h. BP: bovine plasma, CP: corn protein, WP: whey protein. T = effect of time, P = effect of protein treatment, I = interaction time x protein treatment. ^1^n = 33 for BP, n = 34 for CP, n = 30 for WP. ^2^n = 34 for BP, n = 35 for CP n = 32 for WP.^32^n = 36 for BP, n = 36 for CP n = 35 for WP.

### 3.4 PAA uptake kinetics

**Fig. 2:**
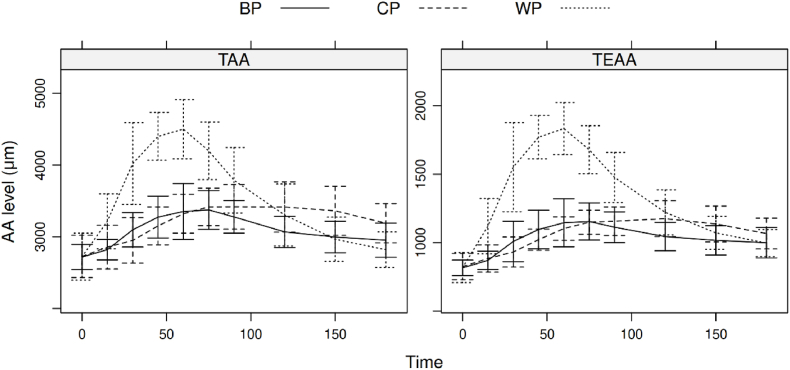
Changes in free total amino acid (TAA) levels and total essential amino acid (TEAA) levels in blood plasma after intake of bovine protein (BP), corn protein (CP) and whey protein (WP).

A randomly selected subgroup of twelve participants were included in the protein digestion analysis. They received 20 g of protein and blood samples were collected postprandially. In these blood samples the concentration of 19 individual free AA was quantified and analysis was performed on the individual amino acids, total amino acids and total of nine essential amino acids (His, Ile, Leu, Lys, Met, Phe, Thr, Trp and Val). Mean postprandial changes in blood total amino acids and total essential amino acids are shown in Fig. 2.

Highest responses on postprandial free AA concentrations were observed for WP. Three hours after consumption, the PAA concentrations did not fully return to baseline, especially after the BP and CP intervention. Outcomes of the AUC, peak height and time2max for each individual amino acid after each intervention protein product are listed in Supplemental Table 3, 5 and 6. Data per study participant on TAA and TEAA for the AUC values showed a large variation in response between individuals (Supplementary Table 4).

We estimated relative uptake by comparing AUC values of BP and CP to the benchmark WP (set at 100%). TAA uptake for BP was 44% and for CP 61% if compared to WP. For TEAA this was 44% for BP and 51% for CP (Supplementary Table 4). We calculated these AUC values though curve fittings over the sampling period of 180 min. Since the PAA levels after CP consumption did not return to baseline after 180 min, we modified curve fittings from the original data points (up to 180 min) to a total of 300 min (data not shown). That increased the TAA of CP relative to WP from 60.7% to 83.8%.

**Fig. 3:**
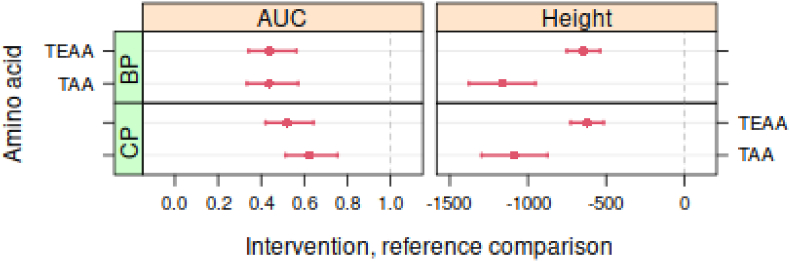
Confidence intervals for total amino acids (TAA) and total essential amino acids (TEAA) when compared to WP as reference comparison. For incremental AUC, the contrast is the ratio between the AUC values of CP and BP intervention and the WP intervention; for the peak height and time2max the contrast is given by the absolute differences (μM or min). Red bars indicate significant differences (P < 0.05) compared to the WP intervention. (For interpretation of the references to colour in this figure legend, the reader is referred to the Web version of this article.)

Statistical comparisons of the AUC, peak height and time2max between interventions were made by using the automatically curated data. Confidence intervals for the TAA and TEAA of these outcomes from the CP and BP intervention were compared to the benchmark WP intervention (Fig. 3). The AUC, peak hight and time2max of the TAA and TEAA were significant lower in the BP and CP if compared to the WP.

**Fig. 4:**
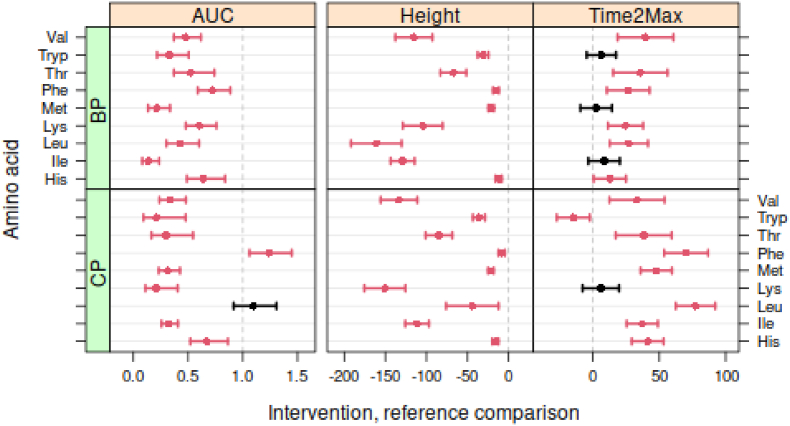
Confidence intervals essential amino acids when compared to WP as reference comparison. For AUC, the contrast is the ratio between the AUC values of CP and BP intervention and the WP intervention; for the peak height and time2max the contrast is given by the absolute differences (μM or min). Red bars indicate significant differences (P < 0.05) compared to the WP intervention. (For interpretation of the references to colour in this figure legend, the reader is referred to the Web version of this article.)

The BP and CP AUC values of most EAA were significantly lower compared to the WP intervention. However, the AUC value of leucine (Leu) of CP was not significantly different from those obtained after the WP intervention (Fig. 4). The AUC values of Phenylalanine (Phe) of CP were higher from those obtained after the WP intervention. Peak height was significant lower for all EAA after the CP and BP interventions compared to WP, while time2max was significant later in time for almost all EAA.

### 3.5 Appetite assessment

Self-assessed scores for hunger, fullness, desire, and appetite changed significantly from before the intake of the protein product to 30–60 min after the intake of the intervention product after which these returned to baseline over the subsequent 2–2.5 h (Supplemental Fig. 3). No significant differences were found in these appetite scores between protein sources.

### 3.6 Cardiometabolic health

Table 4 lists the effects of one-week daily consumption of either CP, BP or WP protein on fasting cardiometabolic parameters. BP intervention resulted in a small but significant increase in systolic blood pressure, diastolic blood pressure, augmentation index and central systolic blood pressure compared to the changes in the CP and WP intervention (P < 0.05 for the interaction time x protein treatment). A small but significant decrease in insulin levels was observed after the CP intervention when compared to the changes in the BP and WP intervention (P = 0.03 for interaction time x protein treatment). No differences between the interventions were observed for glucose levels or heart rate.

**Table 4:** Mean changes in cardiometabolic parameters before and after one week BP, CP or WP intervention. Values are expressed as Mean ± SD.

	Protein intervention						P-value*		
	BP		CP		WP				
	before	after	before	after	before	after	T	P	I
Glucose (mmol/L)	5.3 ± 0.5	5.2 ± 0.5	5.2 ± 0.5	5.3 ± 0.6	5.2 ± 0.4	5.3 ± 0.4	0.16	0.97	0.36
Insulin (uIU/ml)	10 ± 6.9	11.7 ± 6.8	11.5 ± 6.7	9.0 ± 6.6	9.2 ± 7.1	10.3 ± 6.5	0.77	0.50	0.03
Systolic BP (mmHg)	124 ± 18	128 ± 18^b^	124 ± 17	121 ± 19^a^	125 ± 18	122 ± 19^a^	0.49	0.01	<0.01
Diastolic BP (mmHg)	70 ± 9	72 ± 9^b^	71 ± 9	70 ± 10^a^	71 ± 9	69 ± 9^a^	0.36	0.18	<0.01
Heart Rate (bpm)	61 ± 10	60 ± 9	61 ± 10	61 ± 9	61 ± 9	61 ± 10	0.48	0.20	0.22
Augmentation index (%)	21 ± 12	23 ± 12^b^	23 ± 11	22 ± 12^a^	22 ± 12	23 ± 12^a^	0.02	0.33	<0.01
Central systolic BP (mmHg)	115 ± 19	120 ± 19^b^	116 ± 18	113 ± 19^a^	117 ± 19	114 ± 19^a^	0.55	<0.01	<0.01

*calculated by using linear mixed models. BP; blood pressure, CP: corn protein, BP: bovine plasma, WP: whey protein. T = effect of time, P = effect of protein treatment, I = interaction time x protein treatment. ^a,b,c^ Within a row, means without a common superscript differ (P < 0.05).

## 4 Discussion

Protein concentrates and isolated are increasingly consumed and have become an important ingredient in our diets. Here, we evaluated, in a double-blind cross-over intervention trial, tolerance, nutritional quality, and potential health effects of two sustainable protein sources, corn protein (CP) and bovine plasma protein (BP), and compared this with a benchmark whey protein (WP). GI (Self-reported) complaints and intestinal permeability did not differ between the interventions, except for higher self-reported belching and nausea scores during the BP intervention. Clear differences in PAA responses were observed after consumption of the different proteins. One week BP consumption significantly increased blood pressure outcomes compared to the other interventions, and a small decrease in insulin was observed after the CP intervention.

In the current study, tolerance was investigated by taking several parameters into account. Participants reported their stool type and frequency on a daily basis and rated their general health, feelings of bloating, belching, flatulence, nausea, diarrhea and constipation. Stool type and frequency were stable and fitted in a healthy stool pattern during the entire study. GI complaints did not significantly differ between the three protein interventions. However, self-reported belching and nausea scores were significantly higher at the start of the BP intervention and remained high during the intervention if compared to the CP and WP intervention. Based on the above it can be concluded that a repetitive daily intake of 40 g protein during one week did not induce serious GI complaints or changes to stool and were well tolerated. Still, the higher belching and nausea scores during the BP may be a point of concern. But perhaps this can be solved if these proteins are consumed in a food matrix.

Next to the self-reported GI complaints, we also studied intestinal permeability in the duodenum, small intestine, colon and whole intestine, using a multi-sugar test after an acetylsalicylic acid challenge. These permeability markers did not differ between protein interventions, but some did change during the time of the study. Acetylsalicylic acid, also known as aspirin, is a NSAID, often used to treat pain, fever, and inflammation. As is found for many NSAIDs, acetylsalicylic acid increases gut permeability and acetylsalicylic acid is commonly used in clinical studies to augment gut permeability (Farhadi et al., 2008, Marchbank et al., 2008; Lambert et al., 2012). Our participants were subjected to six gut permeability tests in a relatively short period of five weeks and received 2000 mg of acetylsalicylic acid during each test. The increased urinary sugar ratios sucralose/erythritol over time may have been caused by the repetitive nature of our measurement method. To our best knowledge, this multi-sugar acetylsalicylic acid test had never been performed on the same subject so many times in succession and therefore, the effect of such multi doses of NSAID is unknown. Our results indicate that this should be done with caution and a longer wash-out period between interventions may be advised. The decrease in urinary sugar ratios sucrose/rhamnose was mainly due to high baseline values in the third intervention period. We cannot explain this and cannot rule out a potential technical error. Results should therefore be interpreted with caution.

The quality of dietary protein is defined in part by its capacity to provide AA and especially EAA. The PAA profiles of a protein source are therefore important to value its potential for human food application. Here we demonstrate, by comparing the AUC values, that TAA uptake for BP was 44% and for CP 61% compared to WP (set at 100%). For TEAA this was for BP 44% and CP 51% relative to WP, indicating a slightly higher essential amino acids uptake score from CP compared to BP. We previously published a similar PAA uptake study with Lemna protein concentrate and collected literature data on AUC values from other protein sources analysed in a human cross-over design with whey as a benchmark protein (Mes et al., 2021). Both the BP and CP AUC outcomes were below or in the range of casein with reported TEAA uptake ranges of 49–60% compared to whey (Tang et al., 2009; Pennings et al., 2011; Gorissen et al., 2016). Still, a higher actual uptake after CP can be expected since the PAA response after CP consumption did not return to baseline after 180 min. Extrapolation of the PAA curves to 5 h predicted a CP TAA uptake of 83.8% relative to WP. However, predicting such a response after the actual 180 min is challenging, as this will be influenced by gastric emptying and transit time in the small intestine. For future clinical studies on PAA kinetics, we would therefore advise to sample at least up to 300 min. A potential interesting finding of our study is the prolonged elevated postprandial levels of the EAA leucine and phenylalanine after CP consumption. The high leucine content of CP was already described in a publication evaluating amino acid content of various plant-based proteins (Gorissen et al., 2018). Leucine, one of the branched-chain EAA, is valued not only as proteogenic but is also anabolic, serving as a regulator for the postprandial stimulation of muscle protein synthesis. High levels of leucine in the blood can stimulate muscle synthesis and inhibit muscle breakdown (Wilkinson et al., 2013; Gorissen et al., 2018; Takegaki et al., 2020; Zaromskyte et al., 2021).

Recent work from our group tested the digestibility of exactly the same protein sources *in vitro* (Ariëns et al., 2021). Our current findings are not completely in line with outcomes observed *in vitro*. Both *in vivo* and *in vitro* measurements for WP showed a high digestibility. Digestibility for CP appears much lower *in vivo* as compared to *in vitro*, since the *in vitro* method predicted the percentage bioavailable TAA to be 86% ± 5% (*in vivo* 60.7%) and TEAA 85% ± 6% (*in vivo* 51.2%) relative to WP. The relative abundance of amino acids in the digestion filtrate of BP *in vitro* was almost equal compared to WP and therefore much higher compared to the results in the current *in vivo* trial. These discrepancies may be explained by the fact that the *in vitro* analysis outcomes also includes peptides and not only free amino acids. Also *in vivo* plasma amino acid content is not an exact reflection of digestibility (Groen et al., 2015). Alternatively, for both protein sources, the low similarity between *in vivo* and *in vitro* results could be due to clotting of proteins in the stomach and gastric emptying, not simulated in the used *in vitro* model, but known to influence digestibility and uptake of some sources of proteins (Mahe et al., 1996). Therefore static *in vitro* models for protein digestibility and bioavailability, even those based on the INFOGEST consensus model (Minekus et al., 2014; Brodkorb et al., 2019) should be interpreted with caution.

Methodologies that are the gold standard to report protein quality are based on protein digestibility and the protein digestibility-corrected amino acid score (PDCAAS) and more recently the digestible indispensable amino acid score (DIAAS) (Dietary protein quality evaluation in human nutrition, 2013). The PDCAAS of whey protein is generally considered to be 1, which is the highest possible score, and its DIAAS values are even higher (Rutherfurd et al., 2015, Mathai et al., 2017). The PDCAAS score for bovine plasma-derived proteins was 78.5, although differences in protein concentrate preparation could have influenced this result (Montero Castillo et al., 2015). Studies on corn protein revealed PDCAAS scores ranging from 28.7 to 67, indicating that preparation methods significantly impact protein quality (Mendes et al., 2016; Zarkadas et al., 1995). To our best knowledge, neither of these novel sources has been assessed using the DIAAS method. Unfortunately, these PDCAAS and DIAAS methods require animal experiments and do not provide information on between-subject variation in response. Determining and quantifying true amino acid digestibility in humans is rather challenging from a methodological perspective and requires expensive and challenging stable isotope techniques (Trommelen et al., 2021). For this reason, amino acid digestibility in humans has been reported for only a limited range of proteins. Here, we measured PAA concentrations in order to estimate the relative digestibility potential of a protein source. Still, appearance of free blood amino acids does only partially reflect the digestibility of a protein source. The amino acid profile in the blood after intake of a meal is determined by metabolism of splanchnic tissue, passage speed in the intestine, protein uptake/breakdown, and endogenous synthesis (Liu et al., 2019).

Next to tolerance and nutritional quality we also investigated biological function and potential health benefits. We evaluated cardiometabolic markers and observed a significant increase in blood pressure and augmentation index, an estimate for arterial stiffness, after consumption of BP compared to WP and CP. The increase in blood pressure following the BP intervention can most likely be attributed to the product's higher salt content. The BP intervention resulted in an additional daily intake of 8.5 g salt per day, thereby exceeding daily recommendations. To ensure suitability for substantial consumption, reducing the BP salt content is highly recommended. Another observation with potential metabolic consequences was the decrease in fasting insulin levels after the CP intervention compared to the other interventions. Proteins from plant sources that enable the maintenance of the glycemic profile may be of interest in the context of type 2 diabetes, but currently their mechanisms of action are unclear (Costa et al., 2020). Since this is a between-groups comparison, the effect is relatively small, and we didn't corrected for multiple testing, we cannot confidently state that this effect has been initiated by the CP intervention Still, the decrease in insulin after the CP when compared to the other interventions may be important findings that need further evaluation. Dietary protein intake is often associated with satiating effects (Morell and Fiszman 2017), hence we were interested in the effect of the test products on appetite status. We did not find leads that the test proteins affected appetite rating in a different manner.

We evaluated in a comprehensive manner the impact of three different protein sources on tolerance, nutrient quality and health effects but the study had some limitations. We performed a relatively high number of measurements but decided not to apply a correction for multiple testing, because the effect sizes in nutritional interventions are relatively small and we wanted to identify and explore any potential effect. We also had a relative short intervention period of only one week, longer studies are needed to determine the long-term effects. We also do realize that we subjected our study participants to an intensive cross-over study protocol with relatively short wash out periods. This may have influenced some study outcomes, especially the gut permeability test with acetylsalicylic acid challenge.

Consumption of sustainably produced protein in isolated and concentrated form is relatively new, but these proteins are becoming an important factor in our daily diets. Here we demonstrate, for two alternative proteins, that we can consume these protein concentrates in relative high quantities without experiencing unwanted gastro intestinal side effects, that they can be a good source of essential amino acids, while acknowledging that the relative high salt content of the BP may still pose concerns for our health. Future research should focus on longer interventions and implementing these proteins in products and diets that replace conventional proteins to learn more on the health impact when implemented in a regular diet.

## Funding

This project received financial support from the Agri & Food Top Sector program of the Dutch Ministry of Agriculture Nature and Food Quality, and the Sustainable Future Proteins-consortium (TKI-AF 15,269) in collaboration with BASF, Cargill, Coöperate AVEBE U.A., Darling Ingredients, Lesaffre, Marlow Foods, PepsiCo, Roquette, Mimetas B.V., Danone Nutricia Research and Proti-Farm R&D B.V. The views expressed in this manuscript are those of the authors and do not necessarily reflect the position or policy of the collaborating companies.

## Credit Author Statement

Diederik Esser: Design, planning and organization of the trial, Formal analysis and Writing – original draft, Writing – review & editing. Jurriaan Mes: Design of the trial, Formal analysis, Writing – original draft, Writing – review & editing. Kaatje Lenaerts: analysis of the MS in the samples, Writing – review & editing. Ron Wehrens: Formal analysis, mathematics and statistics. Jasper Engel: Formal analysis, mathematics and statistics. Ron T.M. den Dool: Investigation, Formal analysis of amino acids. Shanna Bastiaan-Net Coordination the overall research project, grand, funding and contracts. Harry J. Wichers: Project administration, Funding acquisition, Supervision, Writing – original draft, Writing – review & editing.

## Declaration of competing interest

The authors declare that they have no known competing financial interests or personal relationships that could have appeared to influence the work reported in this paper

## Data Availability

The authors are unable or have chosen not to specify which data has been used.
