# Supplementary material for: Evaluating and comparing tolerance, nutritional quality and bio-functional activity of bovine-plasma, corn and whey proteins, outcomes of a randomized double blind controlled trial
Source: Curr Res Food Sci. 2023 Sep 12;7:100588. doi: 10.1016/j.crfs.2023.100588 (PMC10539881; doi:10.1016/j.crfs.2023.100588)
Supplement: Multimedia component 1 [file mmc1.docx]

# Supplemental tables

**Supplemental Table 1.** Composition of the used CP, BP and WP powders

|  | **Protein (%)** | **Moisture (%)** | **Carbohydrates(%)** | **Fat (%)** | **Ash (%)** | **Salt (%)*** |
| --- | --- | --- | --- | --- | --- | --- |
| BP | 70 | 8 | 0 | 2 | 1 | 15 |
| CP | 85 | 5.5 | 4 | 0.4 | 5 | <1 |
| WP | 80 | 4 | <1 | 5 | 2.8 | <1 |

BP: bovine plasma, CP: corn protein, WP: whey protein, ND: not determined. * Salt (Sodium * 2.5)

**Supplemental Table 2.** Urinary sugar ratios after an acetylsalicylic acid challenge, before and after a one-week CP, BP or WP intervention, divided over the three intervention periods

|  | **Intervention period 1** | | | | | | | | | | |
| --- | --- | --- | --- | --- | --- | --- | --- | --- | --- | --- | --- |
|  | CP | | | | WP | | | BP | | |  |
|  | Count | baseline | endline | Count | | baseline | endline | Count | baseline | endline |  |
| Sucrose/rhamnose (F1) | 12 | 10 ± 15 | 17 ± 22 | 12 | | 8 ± 16 | 10 ± 9 | 12 | 12 ± 22 | 18 ± 23 |  |
| Lactulose/rhamnose (F1) | 12 | 72 ± 33 | 71 ± 25 | 10 | | 64 ± 25 | 56 ± 23 | 12 | 77 ± 61 | 68 ± 53 |  |
| Sucralose/erythritol (F2) | 12 | 26 ± 6 | 21 ± 4 | 12 | | 24 ± 4 | 24 ± 5 | 12 | 25 ± 6 | 24 ± 4 |  |
| Sucralose/erythritol (F1-2) | 12 | 24 ± 7 | 20 ± 4 | 12 | | 23 ± 5 | 23 ± 4 | 12 | 25 ± 4 | 21 ± 3 |  |
|  | **Intervention period 2** | | | | | | | | | | |
|  | CP | | | | WP | | | BP | | |  |
|  | Count | baseline | endline | Count | | baseline | endline | Count | Baseline | Endline |  |
| Sucrose/rhamnose (F1) | 12 | 15 ± 12 | 19 ± 18 | 12 | | 8 ± 5 | 10 ± 8 | 12 | 12 ± 12 | 21 ± 15 |  |
| Lactulose/rhamnose (F1) | 11 | 73 ± 45 | 94 ± 67 | 12 | | 59 ± 33 | 54 ± 26 | 12 | 65 ± 51 | 63 ± 28 |  |
| Sucralose/erythritol (F2) | 12 | 17 ± 6 | 29 ± 10 | 12 | | 19 ± 7 | 26 ± 8 | 12 | 17 ± 7 | 29 ± 8 |  |
| Sucralose/erythritol (F1-2) | 12 | 17 ± 6 | 28 ± 8 | 12 | | 17 ± 6 | 25 ± 8 | 12 | 16 ± 6 | 26 ± 7 |  |
|  | **Intervention period 3** | | | | | | | | | | |
|  | CP | | | | WP | | | BP | | |  |
|  | Count | baseline | endline | Count | | baseline | endline | Count | baseline | endline |  |
| Sucrose/rhamnose (F1) | 10 | 43 ± 27 | 8 ± 5 | 6 | | 43 ± 20 | 15 ± 13 | 9 | 46 ± 23 | 5 ± 3 |  |
| Lactulose/rhamnose (F1) | 12 | 55 ± 19 | 53 ± 25 | 10 | | 70 ± 46 | 79 ± 41 | 10 | 59 ± 37 | 25 ± 25 |  |
| Sucralose/erythritol (F2) | 12 | 14 ± 5 | 23 ± 6 | 11 | | 15 ± 4 | 27 ± 5 | 12 | 17 ± 5 | 27 ± 5 |  |
| Sucralose/erythritol (F1-2) | 12 | 13 ± 3 | 23 ± 4 | 11 | | 16 ± 5 | 26 ± 8 | 12 | 15 ± 4 | 27 ± 5 |  |

Mean ± SD, F2: urinary fraction 5-24 hours. BP: bovine plasma, CP: corn protein, WP: whey protein.

**Supplemental Table 3*.*** Area under the curve (AUC) of the individual amino acid concentrations after consumption of the BP, CP and WP. Area under the curve expressed in arbitrary units.

| **Amino acid** | **BP** | **CP** | **WP** |  | **P-Value** |
| --- | --- | --- | --- | --- | --- |
| Ala | 360 ± 186^a^ | 360 ± 184^a^ | 1192 ± 399^b^ |  | <0.01 |
| Asn | 104 ± 48^a^ | 157 ± 55^a^ | 263 ± 78^b^ |  | <0.01 |
| Asp | 6 ± 2^a^ | 3 ± 2^a^ | 17 ± 13^b^ |  | <0.01 |
| Cys | 141 ± 72^a^ | 63 ± 25^a^ | 299 ± 187^b^ |  | <0.01 |
| Gln/Arg | 837 ± 249^a^ | 1194 ± 432^b^ | 1287 ± 357^b^ |  | <0.01 |
| Glu | 73 ± 38^a^ | 142 ± 53^b^ | 132 ± 61^b^ |  | <0.01 |
| Gly | 175 ± 73^a^ | 144 ± 80^a^ | 342 ± 128^b^ |  | <0.01 |
| His | 84 ± 29^a^ | 95 ± 40^a^ | 137 ± 34^b^ |  | <0.01 |
| Ile | 123 ± 41^a^ | 288 ± 72^b^ | 893 ± 142^c^ |  | <0.01 |
| Leu | 605 ± 225^a^ | 1526 ± 410^b^ | 1440 ± 255^b^ |  | <0.01 |
| Lys | 554 ± 227^b^ | 185 ± 64^a^ | 897 ± 170^c^ |  | <0.01 |
| Met | 32 ± 12^a^ | 48 ± 19^a^ | 156 ± 33^b^ |  | <0.01 |
| Phe | 126 ± 45^a^ | 208 ± 58^b^ | 174 ± 30^b^ |  | <0.01 |
| Pro | 241 ± 170^a^ | 692 ± 246^b^ | 613 ± 165^b^ |  | <0.01 |
| Ser | 210 ± 106^a^ | 223 ± 82^a^ | 367 ± 93^b^ |  | <0.01 |
| Thr | 354 ± 152^a^ | 213 ± 75^a^ | 683 ± 209^b^ |  | <0.01 |
| Tryp | 88 ± 40^a^ | 50 ± 16^a^ | 254 ± 73^b^ |  | <0.01 |
| Tyr | 179 ± 77^a^ | 276 ± 113^b^ | 285 ± 83^b^ |  | 0.01 |
| Val | 649 ± 253^a^ | 436 ± 137^a^ | 1307 ± 293^b^ |  | <0.01 |

*Mean ± standard deviations, calculated by using the automatically curated data. ^a,b,c^ Within a row, means without a common superscript differ (P<0.05).

**Supplemental Table 4.** Area under the curve (AUC) of total amino acids (TAA) and total essential amino acids (TEAA) for the 12 subjects after consumption of the BP, CP and WP.

|  | **TAA** | | | **TEAA** | | |
| --- | --- | --- | --- | --- | --- | --- |
| **Subjects** | **BP** | **CP** | **WP** | **BP** | **CP** | **WP** |
| 1 | 2867 | 3543 | 11158 | 1678 | 1784 | 6838 |
| 2 | 1869 | 3125 | 8037 | 749 | 1476 | 4593 |
| 3 | 3918 | 8692 | 9992 | 1740 | 3746 | 5861 |
| 4 | 3602 | 7277 | 8600 | 2802 | 3237 | 5700 |
| 5 | 6791 | 7607 | 11623 | 3857 | 3289 | 5907 |
| 6 | 5899 | 4519 | 10687 | 3305 | 2338 | 5675 |
| 7 | 3704 | 3967 | 10068 | 2273 | 2418 | 5563 |
| 8 | 5087 | 7203 | 12775 | 2557 | 3284 | 6880 |
| 9 | 3965 | 7524 | 13254 | 2300 | 3579 | 7268 |
| 10 | 3345 | 5476 | 7696 | 2226 | 2989 | 4239 |
| 11 | 7640 | 8047 | 9091 | 4378 | 3970 | 6133 |
| 12 | 3874 | 6184 | 7592 | 2798 | 3141 | 4252 |
| Mean | 4380 | 6097 | 10048 | 2555 | 2938 | 5742 |
| stdev | 1668 | 1913 | 1918 | 984 | 775 | 993 |
| CV | 38 | 31 | 19 | 39 | 26 | 17 |
| Mean relative to WP (%) | 44 | 61 | 100 | 44 | 51 | 100 |

**Supplemental Table 5.** Mean peak height of the individual amino acid concentrations after consumption of the BP, CP and WP intervention*. Data expressed as µM.

| **Amino acids** | **BP** | **CP** | **WP** |  | **P-Value** |
| --- | --- | --- | --- | --- | --- |
| Ala | 76 ± 32^a^ | 56 ± 21^a^ | 212 ± 59^b^ |  | <0.01 |
| Asn | 18 ± 8^a^ | 20 ± 7^a^ | 57 ± 13^b^ |  | <0.01 |
| Asp | 1 ± 1^a^ | 1 ± 1^a^ | 6 ± 4^b^ |  | <0.01 |
| Cys | 19 ± 8^b^ | 8 ± 3^a^ | 46 ± 15^c^ |  | <0.01 |
| Gln/Arg | 132 ± 46^a^ | 144 ± 44^a^ | 239 ± 52^b^ |  | <0.01 |
| Glu | 10 ± 4^a^ | 21 ± 7^b^ | 31 ± 12^c^ |  | <0.01 |
| Gly | 35 ± 12^a^ | 29 ± 10^a^ | 81 ± 28^b^ |  | <0.01 |
| His | 15 ± 5^a^ | 13 ± 4^a^ | 28 ± 4^b^ |  | <0.01 |
| Ile | 21 ± 8^a^ | 37 ± 9^b^ | 150 ± 23^c^ |  | <0.01 |
| Leu | 76 ± 24^a^ | 193 ± 52^b^ | 240 ± 35^c^ |  | <0.01 |
| Lys | 80 ± 29^b^ | 32 ± 8^a^ | 183 ± 35^c^ |  | <0.01 |
| Met | 6 ± 2^a^ | 6 ± 2^a^ | 28 ± 5^b^ |  | <0.01 |
| Phe | 17 ± 5^a^ | 25 ± 7^b^ | 33 ± 6^c^ |  | <0.01 |
| Pro | 35 ± 18^a^ | 85 ± 29^b^ | 116 ± 27^c^ |  | <0.01 |
| Ser | 30 ± 14^a^ | 28 ± 10^a^ | 79 ± 19^b^ |  | <0.01 |
| Thr | 42 ± 17^a^ | 26 ± 8^a^ | 110 ± 23^b^ |  | <0.01 |
| Tryp | 14 ± 5^a^ | 8 ± 2^a^ | 44 ± 10^b^ |  | <0.01 |
| Tyr | 24 ± 10^a^ | 34 ± 13^a^ | 47 ± 12^b^ |  | <0.01 |
| Val | 76 ± 27^a^ | 55 ± 13^a^ | 189 ± 30^b^ |  | <0.01 |

*Mean ± standard deviations, calculated by using the automatically curated data. ^a,b,c^ Within a row, means without a common superscript differ (P<0.05).

**Supplemental Table 6.** Mean Time-2-max of individual amino acid concentrations after consumption of the BP, CP and WP intervention*. Time2max is expressed in minutes.

| **Amino acid** | **BP** | **CP** | **WP** |  | **P-Value** |
| --- | --- | --- | --- | --- | --- |
| Ala | 56 ± 12^a^ | 90 ± 28^b^ | 53 ± 11^a^ |  | <0.01 |
| Asn | 62 ± 11^a^ | 101 ± 19^b^ | 49 ± 10^a^ |  | <0.01 |
| Asp | 83 ± 50^a^ | 87 ± 32^a^ | 48 ± 12^a^ |  | 0.33 |
| Cys | 99 ± 38^b^ | 105 ± 32^b^ | 69 ± 11^a^ |  | <0.01 |
| Gln/Arg | 65 ± 10^b^ | 94 ± 20^c^ | 51 ± 9^a^ |  | <0.01 |
| Glu | 89 ± 59^b^ | 117 ± 22^b^ | 56 ± 13^a^ |  | <0.01 |
| Gly | 54 ± 13^b^ | 72 ± 25^b^ | 40 ± 7^a^ |  | <0.01 |
| His | 66 ± 11^b^ | 87 ± 18^c^ | 50 ± 9^a^ |  | <0.01 |
| Ile | 64 ± 13^a^ | 92 ± 16^b^ | 55 ± 11^a^ |  | <0.01 |
| Leu | 88 ± 24^b^ | 132 ± 18^c^ | 57 ± 11^a^ |  | <0.01 |
| Lys | 75 ± 19^b^ | 58 ± 24^a^ | 49 ± 10^a^ |  | <0.01 |
| Met | 51 ± 7^a^ | 96 ± 20^b^ | 49 ± 10^a^ |  | <0.01 |
| Phe | 72 ± 17^b^ | 113 ± 24^c^ | 46 ± 10^a^ |  | <0.01 |
| Pro | 62 ± 15^a^ | 134 ± 25^b^ | 50 ± 10^a^ |  | <0.01 |
| Ser | 74 ± 20^b^ | 93 ± 22^b^ | 48 ± 10^a^ |  | <0.01 |
| Thr | 97 ± 32^b^ | 96 ± 22^b^ | 58 ± 12^a^ |  | <0.01 |
| Tryp | 68 ± 12^b^ | 51 ± 25^a^ | 60 ± 11^b^ |  | <0.01 |
| Tyr | 83 ± 20^b^ | 124 ± 20^c^ | 56 ± 10^a^ |  | <0.01 |
| Val | 103 ± 34^b^ | 91 ± 16^b^ | 59 ± 12^a^ |  | <0.01 |

*Mean ± standard deviations, calculated by using the automatically curated data. ^a,b,c^ Within a row, means without a common superscript differ (P<0.05).


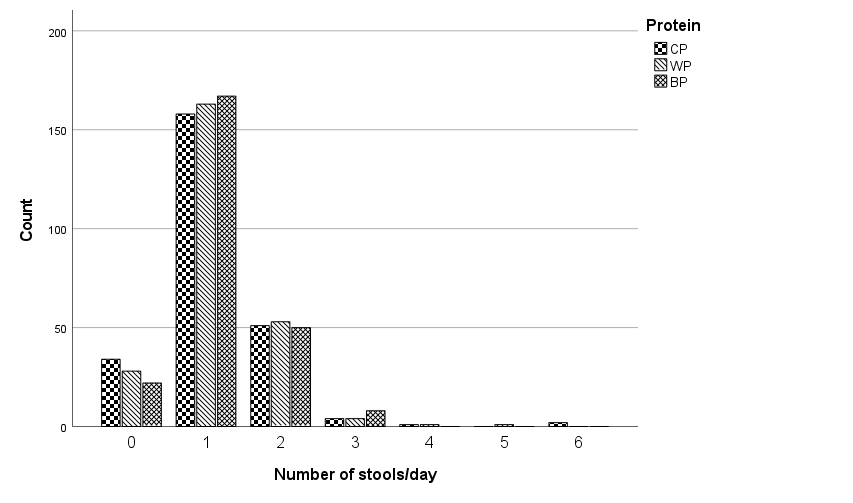


**Supplemental Figure 1.** Counts of number of stools per day during the BP, CP and WP interventions.


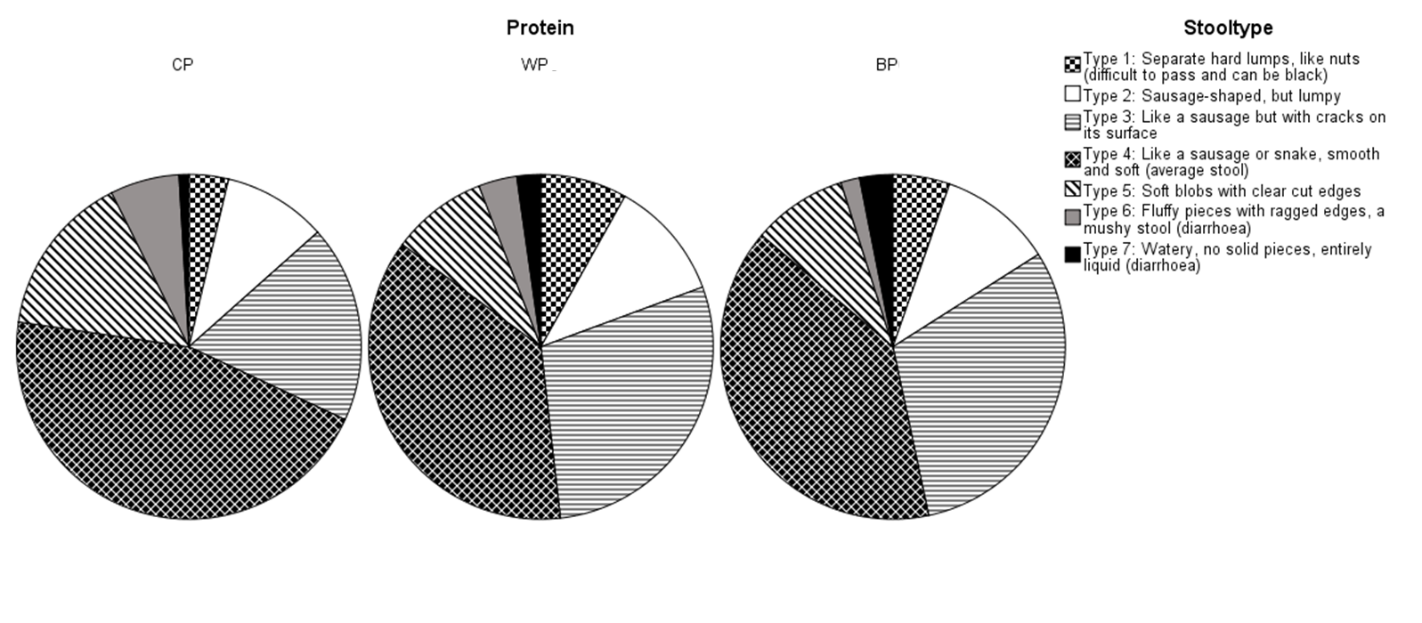


**Supplemental Figure 2.** Relative distribution of stool types based on the Bristol stool charge during the BP, CP and WP intervention. In case of multiple stools during a day, the first stool was scored.

**
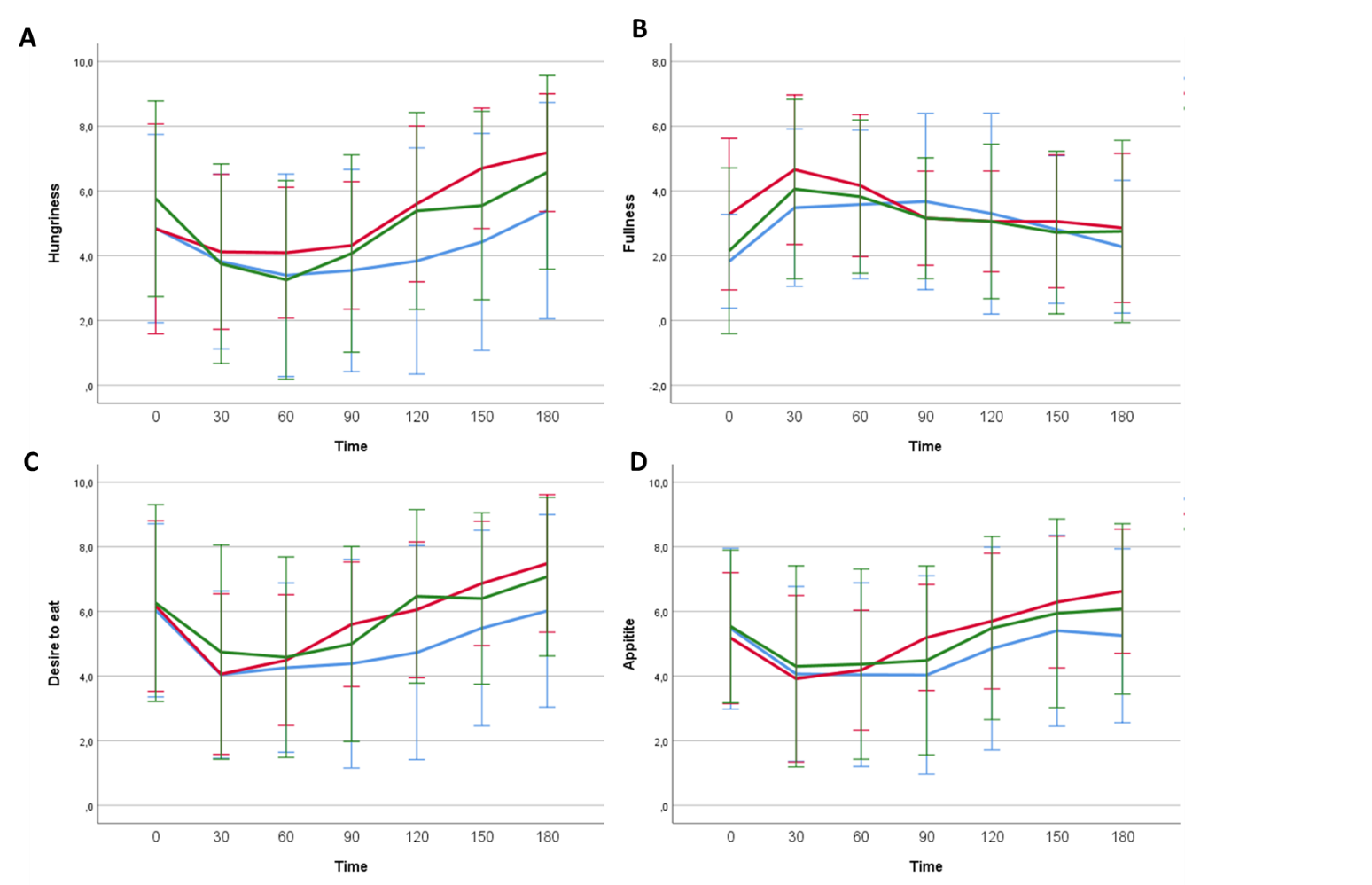
**

**Supplemental Figure 3.** Self-assessed scoring of appetite status over time (in minutes) after BP (green) ,CP (blue) and WP (red) consumption. Scores were anchored at 0 ‘not at all’ and at 10 ‘a lot’. (A) Hungriness score in response to ‘How hungry do you feel?’. (B) Fullness score in response to ‘How full do you feel?’. (C) Desire to eat score in response to ‘How much would you like to eat something right now?’. (D) Appetite score in response to ‘How much do you think you can eat?’. Markers indicate mean ± SD (n=12). There was an effect of time on all qualities of status, but no protein × time interactions.
